# On Agitation

**Published:** 1920-12-25

**Authors:** 


### ON AGITATION.

To many people possessing early Victorian minds the words agitator and anarchist are synonymous. I once heard a worthy hospital matron declare that she invariably took steps to get rid of a nurse who was an agitator, a statement made without qualification or reference to any possible cause of complaint. Being uttered, as it was, to hospital governors, it was warmly approved. The matron in question was in every sense a success—an efficient nurse, a reasonable disciplinarian, and, withal, popular with her staff. An agitator she would not keep on the premises, forgetful, no doubt, that one of the most active and successful agitators of the old generation was Florence Nightingale. I am afraid that the attitude is typical. It may not represent all hospital matrons, but probably the great majority.

There is, however, a world of difference between the office and duties of a hospital matron, as such, and those of a member of the College Council, and if one and the same person occupy the two positions it is quite obvious that she should possess a dual personality. If she is Mr. Hyde while acting as matron, she should transform into Dr. Jekyll when she sits in council with her fellow members. Otherwise it is quite impossible for her to act as advocate and reformer for her profession.

It is extremely important that this should be realised and admitted. The comparative impotence of the College, like many other evils, finds its origin in a lack of clear thinking. Take the question of pay. It was for a long time evident to everybody that the hospital nurse was underpaid. The College Council took the matter up only after a long delay, and then with noticeable reluctance and half-heartedly. A Committee of Inquiry reported, after investigation, but no pressure was brought to bear on hospital Boards to get them to recognise the recommendations.

What did I expect? I expected, once more, hum. I expected to see appointed interviewing committees whose office it would be to wait on hospital Boards, pressing the case on their attention. I expected to see the result published in papers like THE HOSPITAL week by week, so that the nurse and the public interested in nursing could watch the progress of this much-needed agitation. I foresaw the difficulty of matrons themselves acting on these committees, but I saw no difficulty in the way of their selecting others to do so. There are scores of public women whose services are always available to help their sisters to betterment in an economic struggle.

I cite the case of nurses' pay merely as an illustration. It illustrates the arrival and the passing of a golden opportunity—one which I much fear is now irrevocably lost. The nurse numbered her friends by tens of thousands after the war. Every wounded soldier's mother owed and acknowledged a debt to the hospital nurse. In royal palaces the nurse had scores of all-powerful supporters. All this the College Councillors knew. But they refrained from any activity other than tabulating salary scales and giving them the college imprimatur.

This is not agitation. It is the reverse of agitation. It is the *laisser-faire* principle, or what is more popularly known as "wait and see."

Now to come to a question which is exercising the College—that of voting—may I ask how is a nurse to know for whom to vote? Lord Knutsford says no nurse can possibly know, and he is right. At all events, he was right up to the moment when he announced that the College is too young to accomplish anything. If I were a voter I should regard this as a hint as to Lord Knutsford's policy. But the candidates never publish a policy or set out why they are offering themselves as candidates. Their names may be household words as matrons. It is all dark as to what their attitude is or will be as College Councillors. If the Council want to keep alive and to fulfil any more useful function than organising social gatherings they will remedy this.

If they show that they mean business, then the College voting-papers may be used for other purposes than lighting cigarettes.

IERNE.

